# A deep learning-based automatic staging method for early endometrial cancer on MRI images

**DOI:** 10.3389/fphys.2022.974245

**Published:** 2022-08-30

**Authors:** Wei Mao, Chunxia Chen, Huachao Gao, Liu Xiong, Yongping Lin

**Affiliations:** ^1^ School of Optoelectronic and Communication Engineering, Xiamen University of Technology, Xiamen, Fujian, China; ^2^ Department of Radiology, Fujian Maternity and Child Health Hospital, Fuzhou, Fujian, China

**Keywords:** automatic staging method, tumor segmentation, early endometrial cancer, deep learning, medical image processing

## Abstract

Early treatment increases the 5-year survival rate of patients with endometrial cancer (EC). Deep learning (DL) as a new computer-aided diagnosis method has been widely used in medical image processing which can reduce the misdiagnosis by radiologists. An automatic staging method based on DL for the early diagnosis of EC will benefit both radiologists and patients. To develop an effective and automatic prediction model for early EC diagnosis on magnetic resonance imaging (MRI) images, we retrospectively enrolled 117 patients (73 of stage IA, 44 of stage IB) with a pathological diagnosis of early EC confirmed by postoperative biopsy at our institution from 1 January 2018, to 31 December 2020. Axial T2-weighted image (T2WI), axial diffusion-weighted image (DWI) and sagittal T2WI images from 117 patients have been classified into stage IA and stage IB according to the patient’s pathological diagnosis. Firstly, a semantic segmentation model based on the U-net network is trained to segment the uterine region and the tumor region on the MRI images. Then, the area ratio of the tumor region to the uterine region (TUR) in the segmentation map is calculated. Finally, the receiver operating characteristic curves (ROCs) are plotted by the TUR and the results of the patient’s pathological diagnosis in the test set to find the optimal staging thresholds for stage IA and stage IB. In the test sets, the trained semantic segmentation model yields the average Dice similarity coefficients of uterus and tumor on axial T2WI, axial DWI, and sagittal T2WI were 0.958 and 0.917, 0.956 and 0.941, 0.972 and 0.910 respectively. With pathological diagnostic results as the gold standard, the classification model on axial T2WI, axial DWI, and sagittal T2WI yielded an area under the curve (AUC) of 0.86, 0.85 and 0.94, respectively. In this study, an automatic DL-based segmentation model combining the ROC analysis of TUR on MRI images presents an effective early EC staging method.

## Introduction

Endometrial cancer (EC) is one of the most common malignant diseases worldwide. Its incidence rate increases with the gradual aging of the population and the increase in obesity (Amant et al., 2005). Cancer of the uterine corpus is often referred as EC because more than 90% of cases occur in the endometrium (lining of the uterus) (American Cancer Society, 2021). For EC, the prognosis of patients in the early stage is relatively optimistic. In contrast, the prognosis of EC is extremely poor in the advanced stage due to the metastasis of cancer cells in the body (Amant et al., 2005; Guo et al., 2020). According to the 2020 global cancer statistics (Sung et al., 2021), uterine corpus cancer is the sixth most commonly diagnosed cancer in women, with 417,000 new cases and 97,000 deaths in 2020. According to the American cancer society’s 2021 annual and cancer statistics 2021 report (American Cancer Society, 2021; Siegel et al., 2021), an estimated 66,570 cases of the uterine corpus cancer will be diagnosed and 12,940 women will die from the disease in Unite States. The EC incidence rate increases about 1% per year since the mid-2000s.

According to the International Federation of Gynecology and Obstetrics (FIGO) staging, Stage IA of EC is determined by the tumor invading less than 50% of the myometrium while stage IB of EC is presented as the tumor involving 50% or more of the myometrium (Pecorelli, 2009). The 5-year survival rate for EC stage IA patients after surgery is 90–96% and 78–87% for EC stage IB patients (Haldorsen and Salvesen, 2016; Rezaee et al., 2018; Boggess et al., 2020; Mirza, 2020). And the 5-years survival rate was about 92.6% when the myometrial invasion was less than 50%, and only about 66.0% when the myometrial invasion was greater than 50% (Pinar, 2017). Low-risk patients were discouraged from adjuvant radiation therapy and required only a simple hysterectomy, while high-risk patients usually required adjuvant radiation therapy and were recommended pelvic and para-aortic lymphadenectomy (Amant et al., 2018). Therefore, an efficient and automatic prediction model for early EC staging, before cancer cells invade and spread, not only can improve diagnostic efficiency but also provides valuable information for clinicians to recommend treatment to patients.

Magnetic resonance imaging (MRI) and contrast-enhanced dynamic MRI are very accurate in the local staging of EC (Manfredi et al., 2004). In 2009, the European Society of Urogenital Radiology (ESUR) issued guidelines for the staging of EC. The new guidelines regard MRI as the preferred imaging modality for assessing the disease severity of newly diagnosed EC patients (Kinkel et al., 2009). MRI is now widely accepted as the first choice for the initial staging of EC (Nougaret et al., 2019). However, there would be big differences in the evaluation results of two different radiologists on the same MRI images (Haldorsen and Salvesen, 2012). The main reason is that the pathological evaluation obtained by MRI mostly depends on the experience of the radiologist (Woo et al., 2017).

In recent years, deep learning (DL) as a new computer-aided diagnosis method has been widely used in the field of image recognition (LeCun et al., 2015; Shin et al., 2016; Chan H. et al., 2020). This method can automatically capture the target area after training on a large number of data sets (Litjens et al., 2017; Chan H. P. et al., 2020). Therefore, DL is widely used in medical image processing such as classification between tumor epithelium and stroma (Du et al., 2018), providing new prognostic biomarkers for cancer recurrence prediction (Wang et al., 2016), computer-aided diagnosis (CAD) of prostate cancer and lung cancer (Serj et al., 2018; Song et al., 2018), automatic segmentation of the left ventricle in echocardiographic images (Kim et al., 2021), and classification of benign and malignant breast tumor and quality inspection in mammography images (Borges Sampaio et al., 2011; Rouhi et al., 2015).

Some DL-based studies have been carried out to assess the EC. Y. Kurata et al. established a U-net model for uterine segmentation of images with uterine diseases (Kurata et al., 2019). The average dice similarity coefficient (DSC) of the model for uterine segmentation is 0.82, which proves that the U-net model has good segmentation performance in uterus segmentation. Besides, its segmentation performance is not affected by uterine diseases. However, the model only segmented the uterus on the sagittal T2WI images which means it has not been further applied to the automatic segmentation of early EC. M. Bonatti et al. presented a DL model to locate EC lesion area and evaluate MI depth (Chen et al., 2020). However, the AUC of their model on the test set was 0.78 which indicates the classification performance of their model is relatively unsatisfactory. The main reason is that only a box was used to locate the suspected lesion and the surrounding normal anatomical structure. E. Hodneland et al. used a three-dimensional CNN to segment the tumor in preoperative pelvic MRI images of EC patients (Hodneland et al., 2021). The median DSCs between the segmentation results of their model and two raters are 0.84 and 0.77, respectively. Nevertheless, they didn’t segment the uterus and evaluate the invasion depth. The results of the previous work were relatively unsatisfactory in terms of segmentation performance and did not further analyze the TUR in relation to early EC staging. Therefore, the purpose of this study is twofold: first, to establish a DL-based semantic segmentation model to automatically segment tumor and uterus on MRI images. Second, to analyze the performance and potential of using TUR as a reference for early EC staging.

## Materials and methods

The Institutional Review Board (IRB) of Fujian maternity and child health hospital in China approved our retrospective study, the requirement for informed consent was waived.

**FIGURE 1:**
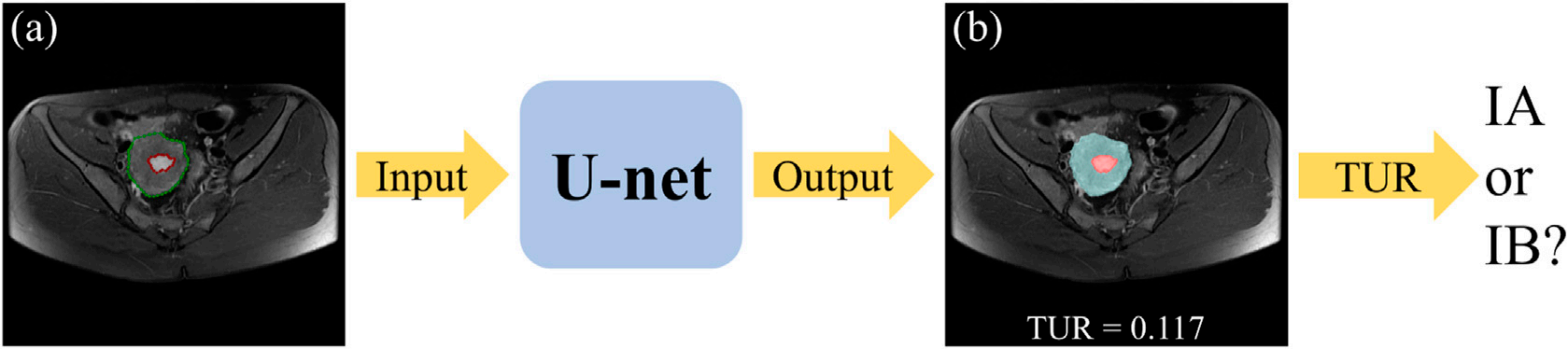
The flowchart of the automatic staging model based on DL. The original MRI image **(A)**, where the red outline is the ground-truth of the tumor and the green outline is the ground-truth of the uterus. A blend of the predicted segmentation map of the U-net and the original MRI image **(B)**, where the red area is the tumor and the blue area is the uterus.

The flowchart of the automatic staging model based on DL is shown in Figure 1. Firstly, both the tumor and the uterus in the input MRI images have been labelled by the experienced radiologist and have been divided into the training set, test set and validation set randomly. Secondly, The U-net segmentation model has been trained by the input training set. Thirdly, the segmentation of the tumor and the uterus in the test set can be obtained by the trained segmentation model. At last, the stage IA or IB of EC patients can be determined by the TUR.

### Patient population

We retrospectively enrolled 117 patients (73 patients in stage IA, 44 patients in stage IB) with a pathological diagnosis of early EC confirmed by postoperative biopsy at our institution (Fujian maternity and child health hospital) from 1 January 2018, to 31 December 2020. All 117 female patients underwent preoperative pelvic MRI and were diagnosed with stage I EC (mean age: 54.8 years, standard deviation (SD): 9.7 years) on postoperative pathology. A summary of the clinical and pathological data of these patients is shown in Table 1.

**TABLE 1:** Clinical and pathological data summaries.

	Patients infomation (*n* = 117)		
Parameter	Stage IA	Stage IB	*p* Value
Subpopulation	73	44	
Age (year)	51.4 ± 8.9	58.9 ± 9.3	0.998
Endometrioid type^*#*^			0.541
Grade 1	49	21	
Grade 2	22	18	
Grade 3	2	5	
Maximum diameter (cm)			0.547
< 3	55	14	
≥3	18	30	
Myometrial invasion			0.748
< 50%	71	3	
≥50%	2	41	
Mixed carcinoma^a^			0.158
No	43	21	
Yes	30	23	

^*#*^ Histological grading of endometrioid carcinoma. According to the solid range of tumors, the classification criteria are as follows: Grade 1: solid growth area ≥5%; Grade 2: the solid growth area accounts for 6% 50%; Grade 3: solid growth area > 50%.

a Indicates the presence of other tumors, such as clear cell carcinoma, uterine fibroids, etc.

### MRI protocol

All MRI examinations were performed on a 1.5 T MRI scanner (Optima MR360, GE Healthcare) with the patients lying supine on the table, with their arms along their bodies. Before the examination, the patient without muscle injection used Glycerini Enema to defecate and suppress the urine. The MRI protocols include: according to the endometrial cavity longest axis, high-resolution T2WI images were acquired along three orthogonal planes (para-axial, para-sagittal) and DWI images were acquired on two planes only (para-axial). All T2WI images are fast spin-echo shimming and the DWI images with B-values of 0, 800 s/*mm*2. The detailed MRI acquisition parameters are listed in Table 2.

**TABLE 2:** Details of parameters for 1.5 Optima MR360 imaging protocols.

Parmameter	Axial T2WI	Axial DWI(b = 0, 800 s/*mm*2)	Sagittal T2WI
Repetition/echo time (msec)	4,000/45	5,000/74	4,200/76
Sequence	SE	EP, SE	SE
Bandwidth (Hz)	162.773	1953.12	122.07
Thickness (mm)	5	5	5
Voxel size (mm)	0.625 × 0.625 × 5	1.25 × 1.25 × 5	0.547 × 0.547 × 5
Rows&Colunms	512 × 512	256 × 256	512 × 512
Flip angle (degrees)	160	90	160

### Data preparation and processing

**FIGURE 2:**
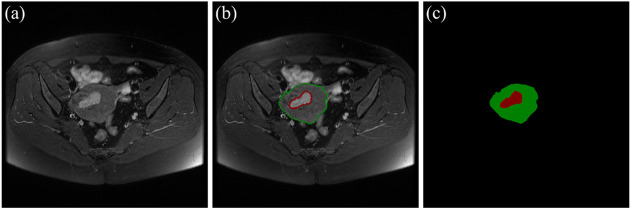
**(A)** The original MRI image. **(B)** The original MRI image with ground-truth contours of the uterus and tumor, where the red outline is the ground-truth of the tumor and the green outline is the ground-truth of the uterus. **(C)** The label image that LabelMe transforms from the ground-truth contours in **(B)** which is used for DL model training.

There are 117 patients with early EC in the data sets, including 73 cases of stage IA and 44 cases of stage IB. An experienced radiologist selected the slices that clearly visualizes the uterus and tumor from each patient’s three different MRI sequences. As a result, 455 MRI images (161 axial T2WI images, 161 axial DWI images, 133 sagittal T2WI images) are obtained. According to the convention of deep learning model training, the dataset is divided in the ratio of 6:1:3 (Andrew, 2018; Jayapandian et al., 2021) The selected MRI images are randomly separated into 70 cases (44 IA/26 IB) including 272 images as the training set, 12 cases (7 IA/5 IB) including 46 images as the validation set, and 35 cases (22 IA/13 IB) including 136 images as the test set. The details of data sets are shown in Table 3. The uterus and tumor on the above MRI images were manually segmented by the experienced radiologist through the software LabelMe (Russell et al., 2008). These segmented areas were used as the ground truth for uterus and tumor segmentation. An original MRI image and the corresponding segmented image are shown in Figure 2.

**TABLE 3:** Details of the data set division.

Type	Training set	Validation set	Test set
Axial T2WI (IA/IB)	99 (61/38)	12 (6/6)	50 (34/16)
Axial DWI (IA/IB)	94 (55/39)	18 (10/8)	49 (33/16)
Sagittal T2WI (IA/IB)	79 (53/26)	16 (9/7)	38 (23/15)
Total (IA/IB)	272 (169/103)	46 (25/21)	137 (90/47)

### Training the DL networks

**FIGURE 3:**
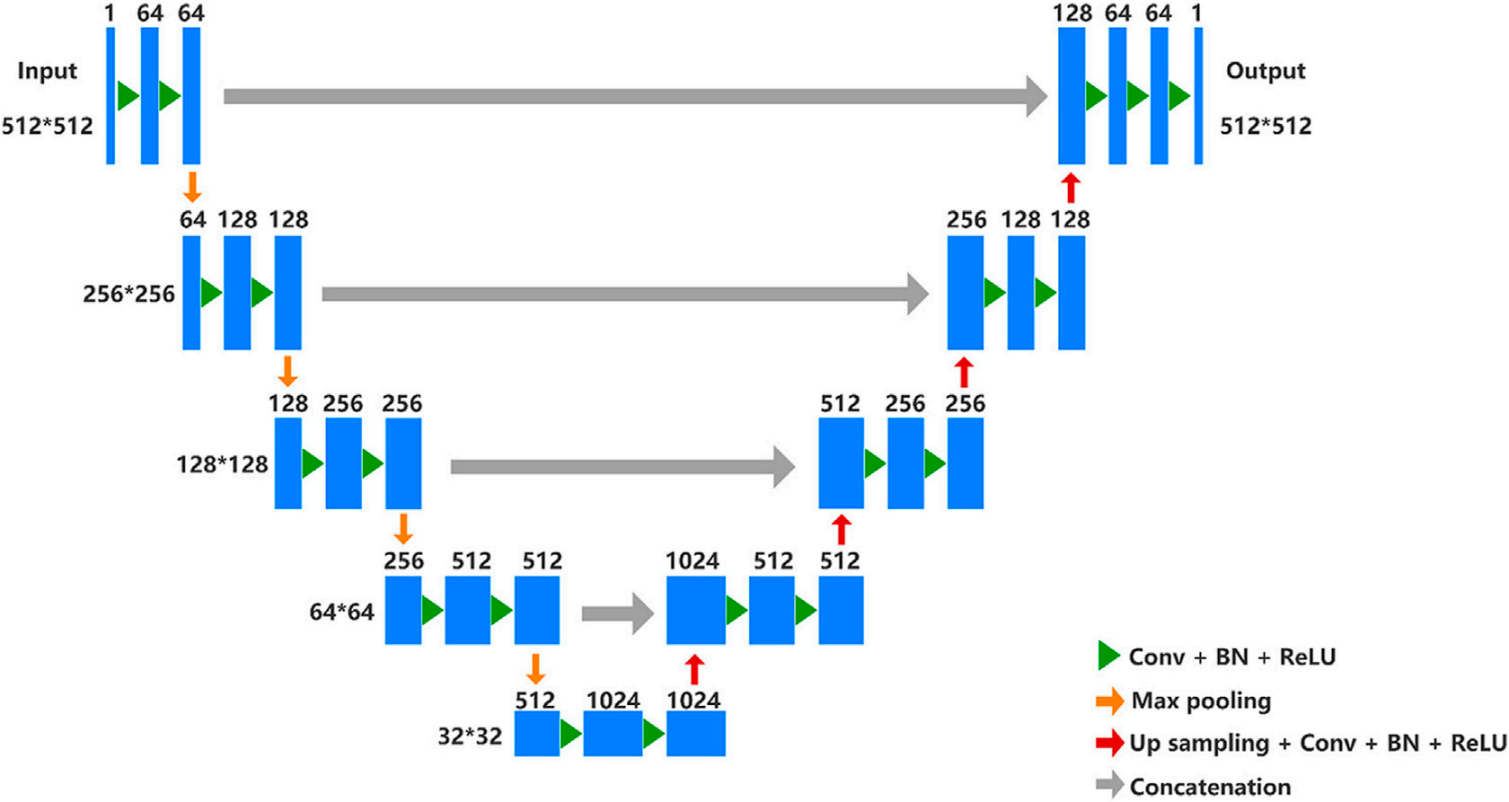
U-net architecture. In the contracting path, every step consists of downsampling with stride two of the feature map and doubles the number of feature channels. Repeated application of two 3 × 3 convolutions (unpadded convolutions), each followed by a rectified linear unit (ReLU) and a 2 × 2 max pooling operation. In the expansive path, every step consists of an upsampling of the feature map followed by a 2 × 2 up convolution that halves the number of feature channels, a concatenation with the correspondingly cropped feature map from the contracting path, and two 3 × 3 convolutions, each followed by a ReLU.

An automatic segmentation DL-based model has been implemented by a U-net architecture to segment the uterus and tumor on the MRI images in this work. U-net is a semantic segmentation network based on fully convolutional networks (FCN), which is suitable for medical image segmentation. The network architecture is shown in Figure 3 and it has both a contraction path to capture context information and an asymmetric expansion path to allow accurate positioning, which makes the network propagate context information to a higher resolution (Ronneberger et al., 2015). The Adam algorithm with an initial learning rate of 0.0003 was used to minimize the cross-entropy loss. The model was implemented by TensorFlow (version 2.5.0) with 300 epochs and employed an early-stop strategy to avoid model overfitting. The experiments were conducted on a workstation equipped with a high-performance graphics processing unit (NVIDIA RTX 2080TI, Gigabyte Ltd.). It took about 3 hours to train the semantic segmentation model.

### Quantitative evaluation of the uterus and tumor segmentation

The DSC is used to measure the overlap of two samples. In this study, DSC was been used to quantitatively evaluate the segmentation overlap of the uterus and the tumor in the test set. DSC is calculated as follows:

$$DSC=\frac{2|X\cap Y|}{\left|X|+|Y\right|}$$ (1)

Where *X* is the real artificial segmentation map and *Y* is the predicted segmentation map. The range of DSC values is from 0 to 1. DSC is 0 when the two images are completely non-overlapping and one when the two images are completely overlapping.

### Calculation of TUR

An area-based approach is used to perform TUR calculations on the segmented images predicted by the DL model in the test set. The number of pixels is used to calculate the area. As shown in Figure 1, the tumor and the uterus areas are obtained to calculate the TUR (i.e., calculate the ratio of the number of pixels in the red area to the number of pixels in both the blue area and the red area). For MRI slices with different scanning parameters, the TUR is all calculated in the same way.

### Validation and statistics

The test set with 35 randomly selected patients (22 IA/13 IB) including 137 images (50 Axial T2WI, 49 Axial DWI, 38 Sagittal T2WI) are used to validate the performance of the semantic segmentation model. For the TURs obtained on the predicted segmentation maps on the three different MRI sequence slices, the ROCs were plotted and the AUCs were calculated. For a patient in the test set, the corresponding slices from three different MRI sequences are chosen for predictive segmentation, and then calculate the TURs of the segmentation maps, the final classification results will be determined by the threshold values obtained from the corresponding ROCs. Statistical analyses were performed on SPSS (version 26.0., SPSS Inc.) and *p*-values were obtained by *t*-test. The test datasets and code implementation presented in this study can be found in online repositories (https://github.com/mw1998/Segmentation-Area-Ratio).

## Results

### Performance of the automatic uterus and tumor segmentation model

**FIGURE 4:**
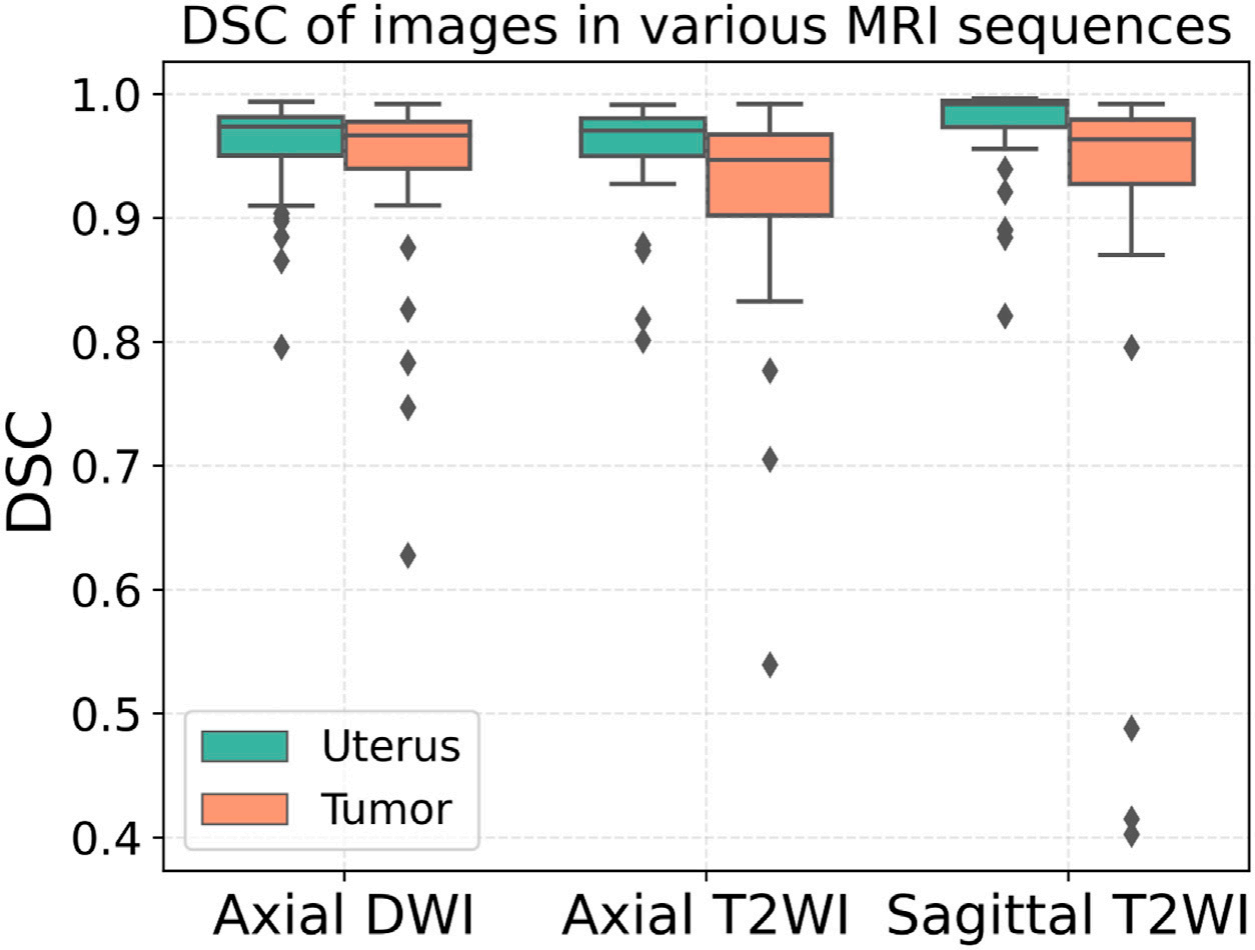
DSCs of the segmented uterus and tumor.

**FIGURE 5:**
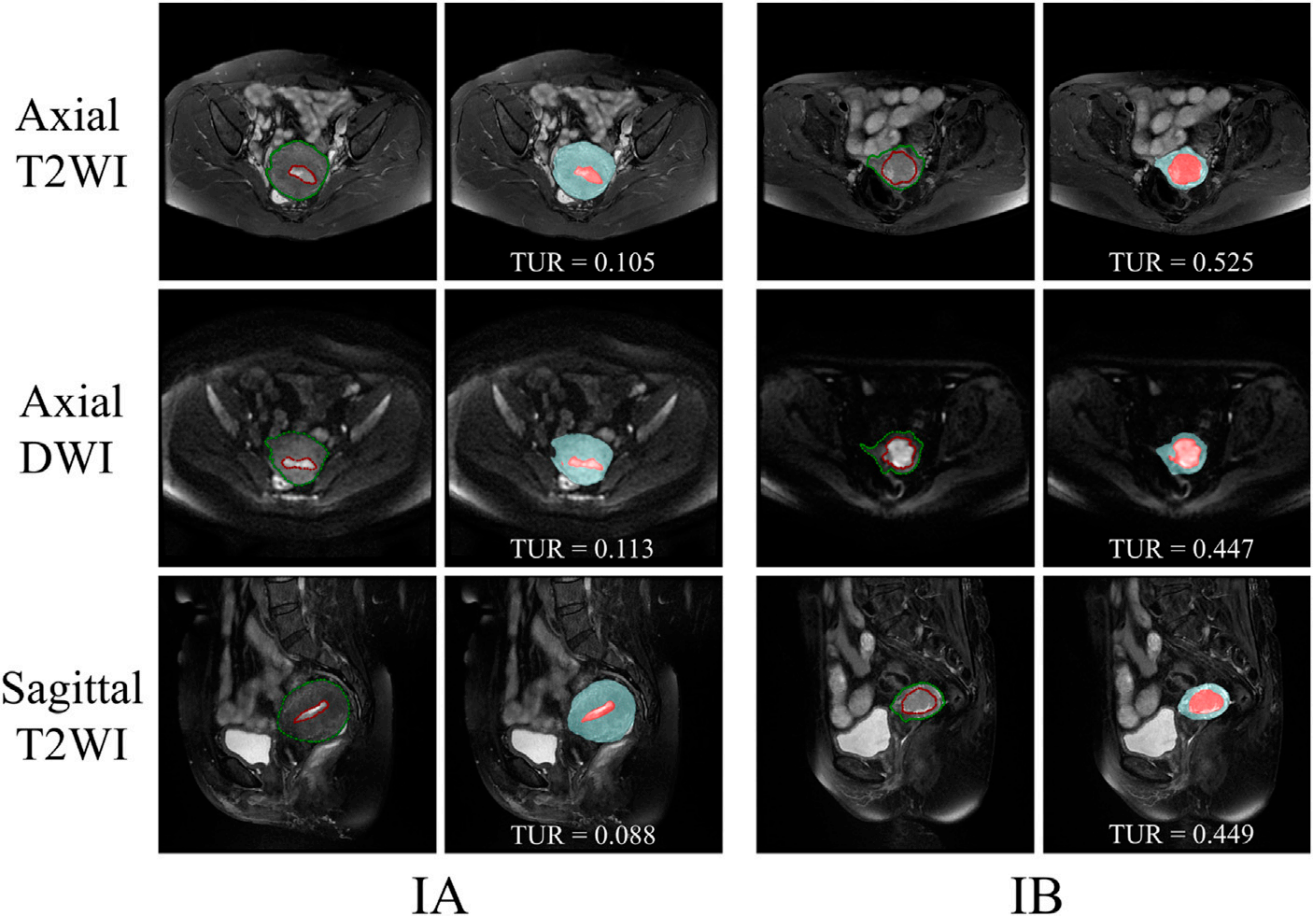
The segmentation results of a patient with EC stage IA (left) and a patient with stage IB (right) on axial T2WI image, axial DWI and sagittal T2WI image. The red outline is the ground-truth of the tumor and the green outline is the ground-truth of the uterus. The red area is the tumor and the blue area is the uterus.

The segmentation results of a patient with EC stage IA (left) and a patient with stage IB (right) on axial T2WI image, axial DWI and sagittal T2WI image are shown in Figure 5. Both the uterus and tumor were well segmented. The average DSC values of uterus and tumor in 137 MRI images in the test set are 0.959 and 0.911, respectively. A box plot of the DSC values is shown in Figure 4). The DSCs of three different MRI images are all over 0.9. As shown in Figure 4, the DSCs of sagittal T2WI images and axial DWI images have better performance in the three MRI sequences, and the DSC of the uterus is obviously higher than that of the tumor. The DSC variances of uterus and tumor in three MRI sequences images are less than 0.15, indicating that the data has little fluctuation and the segmentation model is stable. The DSCs of the segmented uterus and tumor on MRI images are shown in Table 4. The segmented tumor and the segmented uterus are all significantly different (*p* < 0.001) from each other in the three MRI sequences images.

**TABLE 4:** DSCs of the segmented uterus and tumor.

DSC Info	mean ± SD	median	*p* value
All Uterus	0.959 ± 0.089	0.974	< 0.001
All Tumor	0.911 ± 0.123	0.947	
Axial T2WI Uterus	0.964 ± 0.050	0.978	< 0.001
Axial T2WI Tumor	0.918 ± 0.128	0.951	
Axial DWI Uterus	0.952 ± 0.139	0.975	< 0.001
Axial DWI Tumor	0.915 ± 0.143	0.953	
Sagittal T2WI Uterus	0.961 ± 0.023	0.968	< 0.001
Sagittal T2WI Tumor	0.897 ± 0.082	0.923	

### TUR findings

**FIGURE 6:**
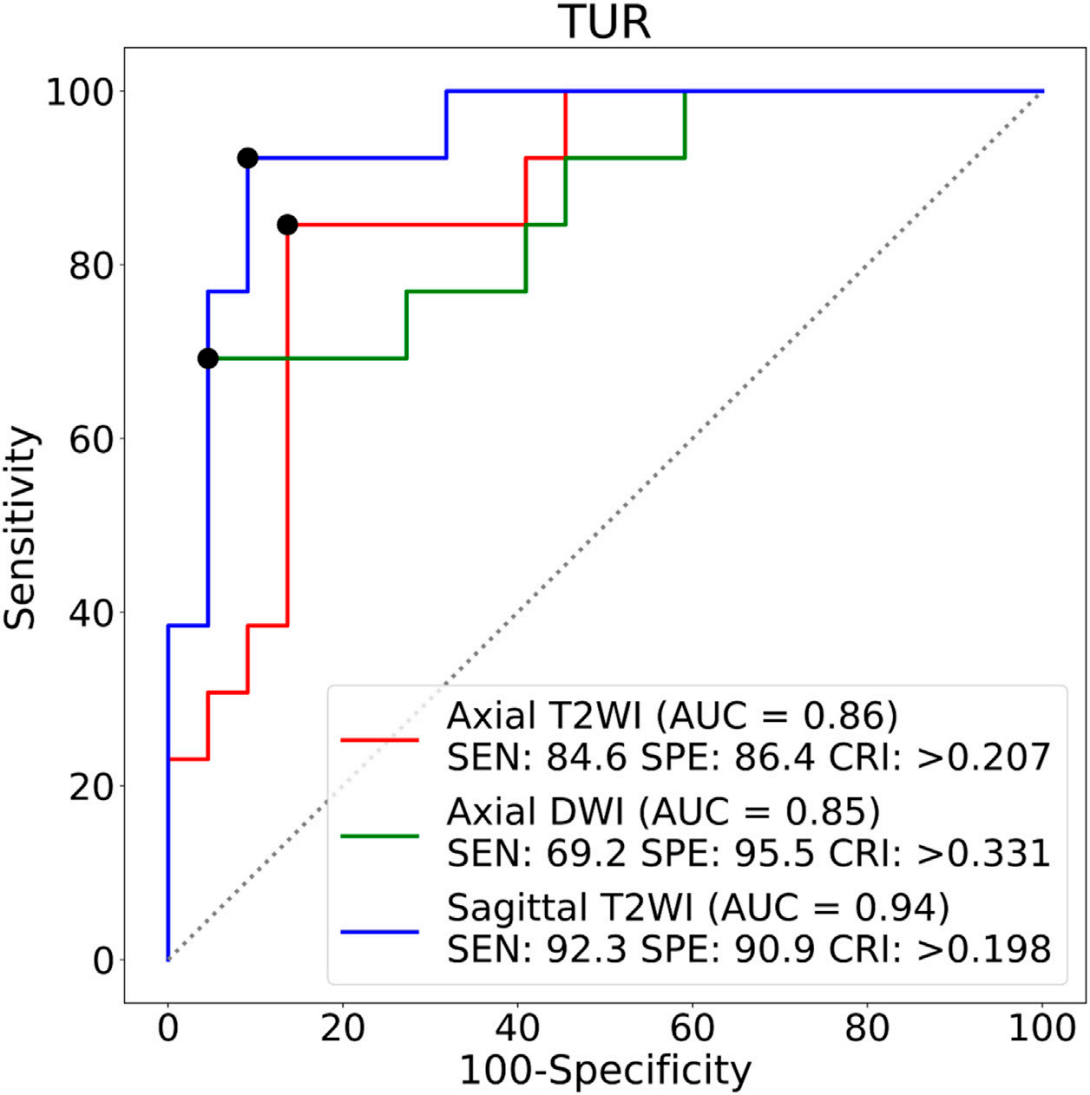
ROCs on three different MRI sequence slices. SEN:Sensitivity, SPE:Specificity, CRI:Criterion.

The TURs of a patient with stage IA and a patient with stage IB on axial T2WI, axial DWI and sagittal T2WI images are shown in Figure 5. Compare to stage IB patient, stage IA patient has small TURs on all three MRI sequence slices. Moreover, small TUR differences in the same stage patients indicate that early EC classification by using TUR is effective. The mean TURs of all stage IA patients and all stage IB patients on three different MRI sequence slices in the test set are shown in Table 5. For axial T2WI images, the mean TUR is 0.165 for stage IA and 0.307 for stage IB. For axial DWI images, the mean TUR is 0.190 for stage IA and 0.335 for stage IB. For sagittal T2WI images, the mean TUR is 0.103 for stage IA and 0.334 for stage IB. All three MRI sequence slices have a statistically significant difference in the stage IA group to the stage IB group. ROCs on three different MRI sequence slices are shown in Figure 6. For axial T2WI images, the threshold of 0.207 can distinguish stage IA from stage IB with 84.6% sensitivity, 86.4% specificity, and AUC of 0.86. For axial DWI images, the threshold of 0.331 can distinguish stage IA from stage IB with 69.2% sensitivity, 95.5% specificity and AUC of 0.85. For sagittal T2WI images, the threshold of 0.198 can distinguish stage IA from stage IB with 92.3% sensitivity, 90.9% specificity and AUC of 0.94.

**TABLE 5:** Mean TURs of all patients on three different MRI sequence slices.

MRI sequence (mean ± SD)	IA	IB	*p* value
Axial T2WI	0.165 ± 0.083	0.307 ± 0.112	< 0.001
Axial DWI	0.190 ± 0.077	0.335 ± 0.117	< 0.001
Sagittal T2WI	0.103 ± 0.077	0.334 ± 0.125	< 0.001

### Comparisons between three different MRI sequence slices

The radiologist decides by viewing more than one MRI sequence slice of an EC patient. Table 6 demonstrates the classification performance of TUR on a single MRI sequence slice and combined different MRI sequence slices for a patient with early EC. The first three rows indicate the classification performance only by one MRI sequence slice. The sagittal T2WI image has the best TUR classification performance, reaching an accuracy of 0.914, a sensitivity of 0.923 and a specificity of 0.909. The middle three rows indicate the classification performance by two MRI sequence slices. The best accuracy is 0.886 by the axial DWI and sagittal T2WI images, while the sensitivity is 1.000 by the axial T2WI and sagittal T2WI images. The last three rows indicate the performance of TUR when using a fuzzy logic approach by at least one MRI sequence, at least two MRI sequences and three MRI sequences. The optimal specificity of 1.000 is obtained by at least one MRI sequence, while an optimal sensitivity of 1.000 is obtained by all three MRI sequences.

**TABLE 6:** The performance of TUR in single MRI sequence and Multiple MRI sequences.

References MRI	Criterion	ACC	SEN	SPE
Axial T2WI	0.207	0.857	0.846	0.864
Axial DWI	0.331	0.857	0.692	0.955
Sagittal T2WI	0.198	0.914	0.923	0.909
Axial T2WI + Axial DWI	—	0.857	0.846	0.864
Axial T2WI + Sagittal T2WI	—	0.857	1.000	0.773
Axial DWI + Sagittal T2WI	—	0.886	0.923	0.864
Least one MRI sequence	—	0.886	0.692	1.000
Least two MRI sequences	—	0.886	0.769	0.955
All	—	0.857	1.000	0.773

## Discussion

In this study, by using a DL-based semantic segmentation model to segment tumor and uterus on three types of MRI images, and then calculate the TUR to classify the stage IA and stage IB for early EC. Employing the patient’s pathological diagnostic results as the gold standard, using TUR for early EC staging has a different performance for different MRI sequence slices. The proposed method has the best performance in classifying early EC on only sagittal T2WI images, yielding an accuracy of 0.914, a sensitivity of 0.923, and a specificity of 0.909. And the classification performance has a higher sensitivity or specificity by multiple MRI sequences. Its main clinical values are: 1. Accurate segmentation of uterus and tumor can help the clinician to better observe the invasion trend of tumor; 2. Using TUR to analyze the tumor invasion of patients can help clinicians to develop more appropriate treatment strategies for patients. For example, while the TUR is small, the clinician may recommend a hysterectomy for the patient. While the TUR is large, the clinician may recommend pelvic and para-aortic lymphadenectomy for the patient. 3. The model is automatic and efficient, which can reduce the clinician’s workload.

**FIGURE 7:**
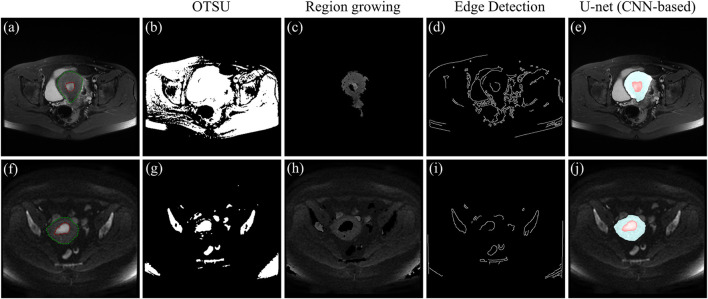
**(A)** and **(F)** MRI images of the test set, where the red outline is the ground-truth of the tumor and the green outline is the ground-truth of the uterus. **(B)** and **(G)** Segmentation results of the OTSU algorithm. **(C)** and **(H)** Segmentation results of the region growth algorithm. **(D)** and **(I)** Segmentation results based on edge detection. **(E)** and **(J)** Segmentation results of the U-net, where the blue area is the uterus region and the red area is the tumor region.

As shown in Figure 7, three traditional machine learning segmentation algorithms are compared with the CNN-based U-net segmentation algorithm on two MRI images of the test set. OTSU is a threshold-based image segmentation algorithm, and the segmentation results of the OTSU algorithm are shown in Figures 7B,G. MRI images are grayscale maps, and because the grayscale values of the uterus and tumor are not significantly different from the grayscale values of other pelvic tissue, the OTSU algorithm was unable to find a threshold for distinguishing both the uterus and tumor from pelvic MRI images. The region growing algorithm is a region-based image segmentation algorithm. The segmentation results of the region growth algorithm are shown in Figures 7C,H. The region growth algorithm requires the initial seed and growth criterion to be set manually according to the image conditions. However, the location and size of the uterus and tumor in each MRI image are different, the region growth algorithm is difficult to be applied to the uterus and tumor segmentation. Figures 7D,I show the preliminary results of the segmentation algorithm based on edge detection. Due to the complex and dense distribution of tissues and organs in the pelvis, there are more edge features in the MRI images. Therefore, the segmentation algorithm based on edge detection is difficult to segment the uterus and tumor from MRI images. Figures 7E,J show the segmentation results of CNN-based U-net on MRI images. It can be seen that U-net segmented the uterus and tumor well on the MRI images and were very close to the region in the ground-truth contour. Compared with traditional machine learning methods, the CNN-based method can automatically and accurately segment both uterus and tumor regions from pelvic MRI images.

(Bonatti et al., 2018) found that the tumor/uterus volume ratio greater than 0.13 was significantly associated with high-grade EC, and the cut-off value of 0.13 enabled to distinguish low-grade EC from high-grade EC with 50% sensibility and 89% specificity. However, volume calculation is time-consuming and has poor utility in daily clinical practice. Besides, there is an error when they use ellipsoidal formulas rather than segmentation to estimate tumor volume. (Hodneland et al., 2021). proposed the use of 3D convolution neural networks for the segmentation of tumors in EC patients on MRI images. The method achieved high segmentation accuracy and accurate volume calculation which are close to the results of radiologists’ manual segmentation. However, accurate manual tumor labelling of 3D image data is highly labor-intensive, and they did not investigate the relationship between the volume ratio of tumor to the uterus and the grade of EC. (Chen et al., 2020). proposed a DL-based two-stage CAD method for assessing the depth of myofilament infiltration of EC on MRI images. The classification method yielded a sensitivity of 0.67, specificity of 0.88, and accuracy of 0.85. However, only T2WI data were used for training the DL network so it could not provide interpretable references to scientists due to the black-box nature of DL. This study differs from previous studies as follows: 1. avoids the use of labor-intensive 3D datasets. 2. analyzes the classification performance of TUR on different MRI sequences for early EC. 3. Compared to using the DL model exclusively, combining the DL model with the TUR analysis method provides a more interpretable reference for the staging of early EC.

**FIGURE 8:**
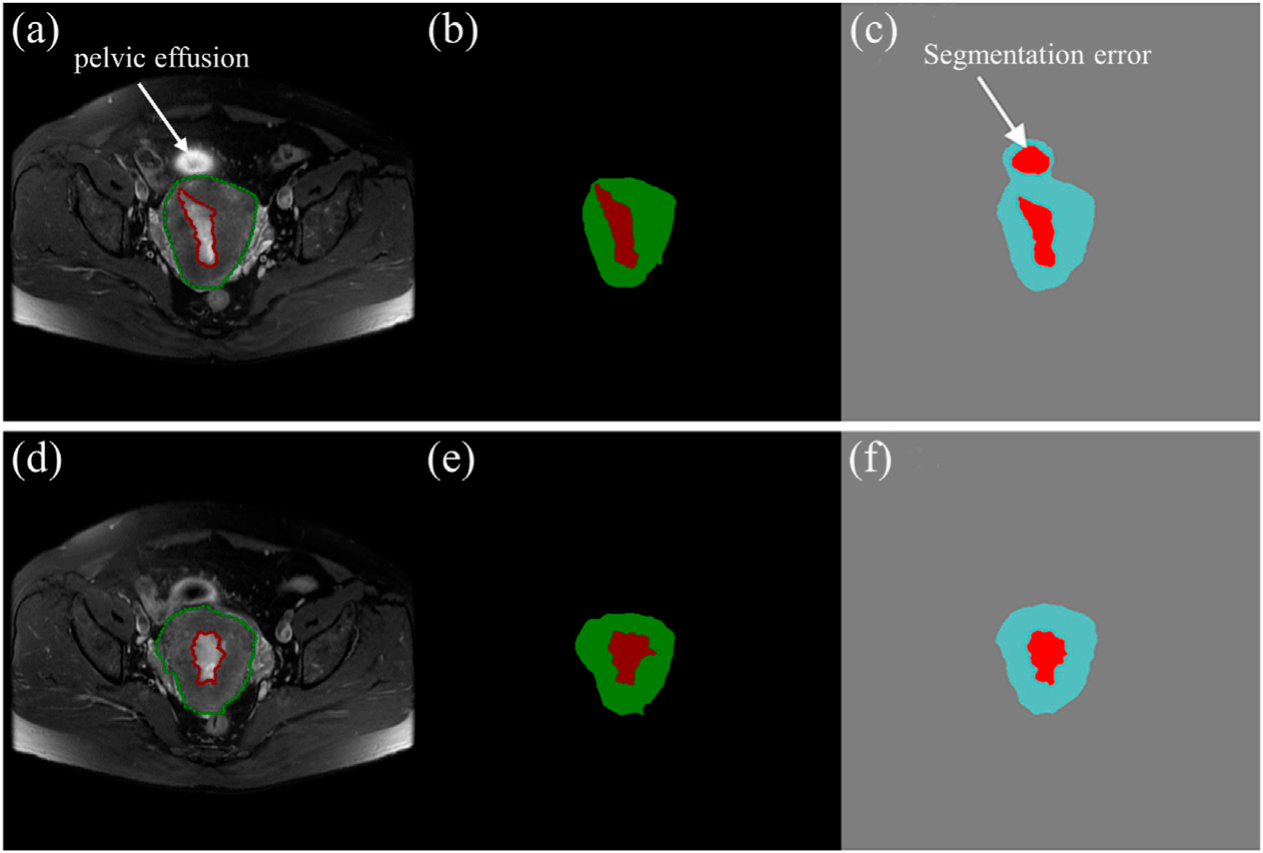
**(A)**, **(D)** The two axial T2WI images from the continuous sequences of a patient. **(B)**, **(E)** The tumor (red) and uterus (green) images labelled by experienced radiologists. **(C)**, **(F)** The predictive tumor (red) and uterus (blue) images by the DL model.

The mean DSCs of the uterus is higher than that of the tumor which is shown in Figure 4. It indicates that the model has a better segmentation effect on the uterus compared to tumors. The main reasons are as follows: The shape of the uterus in the human body is relatively geometrically fixed so that the model can learn the characteristic parameters of the uterus region better. The shape of the tumors in various patterns makes it harder to learn. There are also some interference factors inside or outside the uterus, such as pelvic effusion, hematocele, uterine fibroids, cervical cancer, and so on, which makes the model more complicated in the selection of tumor characteristics parameters. An fault segmentation example is shown (Figure 8). Figure 8A is the original axial T2WI image with a pelvic effusion (pointed by the arrow). The pelvic effusion is false segmented as a tumor by the DL model as shown Figure 8C. The reason could be the brightness similarity of the pelvic effusion and the tumor. Figure 8D is the adjacent image which doesn’t have the bright pelvic effusion like Figure 8A. The tumor is segmented correctly as a tumor as shown Figure 8F. Such kinds of faults will be discussed in future work.

The automatic staging method also has some limitations. Firstly, the number of patient cases used in the study is small and no data of healthy individuals are included. We will improve the model by collecting more patient cases including MRI images of healthy individuals. Secondly, although we have preliminary proved the feasibility of distinguishing stage IA from stage IB by using the method of TUR, the accuracy could also be improved by other methods. Further research will focus on developing a computer-aided diagnosis method that can imitate radiologists’ behavior on early EC staging. Last but not the least, picking the optimal slice from a MRI sequence to be segmented is time-consuming. An automatic picking method would be studied in the future to make the model more practical.

In summary, the results show that the DL-based semantic segmentation model for tumor and uterus segmentation on MRI images and then performing TUR analysis for early EC staging is effective. This method is an automatic and time-saving solution and has the potential to be used for early EC in clinical use.

## Data Availability

The datasets presented in this study can be found in online repositories. The names of the repository/repositories and accession number(s) can be found below: https://github.com/mw1998/Segmentation-Area-Ratio.
